# Evaluation of virtual handles for dental implant manipulation in virtual reality implant planning procedure

**DOI:** 10.1007/s11548-022-02693-1

**Published:** 2022-06-22

**Authors:** Hanna-Riikka Rantamaa, Jari Kangas, Maarit Jordan, Helena Mehtonen, John Mäkelä, Kimmo Ronkainen, Markku Turunen, Osku Sundqvist, Ismo Syrjä, Jorma Järnstedt, Roope Raisamo

**Affiliations:** 1 Faculty of Information Technology and Communication Sciences, Tampere University, Kalevantie 4, 33014 Tampere, Finland; 2 Medical Imaging Centre, Department of Radiology, Tampere University Hospital, Teiskontie 35, 33520 Tampere, Finland; 3 Planmeca, Asentajankatu 6, 00880 Helsinki, Finland

**Keywords:** Virtual dental implant planning, Virtual reality, Dental implant, 3D imaging

## Abstract

****Purpose**:**

Many surgical complications can be prevented by careful operation planning and preoperative evaluation of the anatomical features. Virtual dental implant planning in three-dimensional stereoscopic virtual reality environment has advantages over three-dimensional projections on two-dimensional screens. In the virtual environment, the anatomical areas of the body can be assessed and interacted with in six degrees-of-freedom. Our aim was to make a preliminary evaluation of how professional users perceive the use of the virtual environment on their field.

****Methods**:**

We prepared a novel implementation of a virtual dental implant planning system and conducted a small-scale user study with four dentomaxillofacial radiologists to evaluate the usability of direct and indirect interaction in a planning task.

****Results**:**

We found that all four participants ranked direct interaction, planning the implant placement without handles, to be better than the indirect condition where the implant model had handles.

****Conclusion**:**

The radiologists valued the three-dimensional environment for three-dimensional object manipulation even if usability issues of the handles affected the feel of use and the evaluation results. Direct interaction was seen as easy, accurate, and natural.

## Introduction

In implant surgery, missing teeth are replaced with dental implants and prosthesis, like an implant-supported denture or fixed supra-structures. The workflow of computer-aided design (CAD) includes several steps [1, 2], of which “3-D diagnostics and treatment planning” is of interest here. Implant planning systems based on the three-dimensional (3D) imaging data are standard tools with many systems available [3, 4]. Current systems use the regular two-dimensional (2D) displays as output with a keyboard and a mouse as input devices [5], even as the medical data and the implants are in true 3D. When interaction is done by a mouse, translating and rotating the objects require multiple clicks.

Imaging systems with potential to capture medically and diagnostically relevant images have improved, but devices for visualization and interpretation of the acquired images have lagged in development. The visualization tools used in the medical field are quite conservative compared to tools in the entertainment like 3D movies, augmented reality games, and virtual reality (VR). Radiologists and surgeons usually evaluate 3D images on 2D screens from three standard directions (axial, coronal, and sagittal) [6]. Seeing the object from three angles allows precise manipulation but requires users to have greater spatial understanding [7]. Companies offer software (e.g., NobelGuide, Implant Master, SimPlant, Romexis) for implant planning [1, 8]. The use of these software requires knowledge, and special cross-sectional reconstructions are used.

VR has significant advantages over 3D visualizations on 2D screens. It allows interactivity and immersion within the virtual 3D environment. The interaction in VR is usually done directly with hand controllers. The image can be translated, rotated, and scaled from arbitrary angles [6].

Zorzal et al. [9] studied a VR implant planning system controlled by hand moves and a handheld smartphone device. The system was used by students for learning implant placement. Unlike in [9], we developed our implementation for professional users to compare it with the implant planning systems they were regularly using. Also, Moussa et al. [10] collected a review of VR uses for student’s dental education.

The implants in the VR system are moved to their planned positions by picking them up using the VR controller and doing translations and rotations. While that is straightforward, fine-tuning the position and orientation may be challenging when movements will affect all 6 degrees-of-freedom (6 DoF, 3 position and 3 rotation coordinates) simultaneously. When correcting the position, a small tilt of the controller will also change the orientation.

To facilitate the problem of unwanted movements of linked axes, Mendes et al. [11] used a widget that forced handling the translations and rotations separately, to help in fine-tuning. The translation axes are fixed to the object [11–13], which is easy to understand.

We made a preliminary evaluation of the usability of the implant handles when the participants were asked to position an implant into a suitable location. The task was executed in two conditions, with and without the implant handles. The widget handles will enable more careful moving and turning of the implants but will also use more time as complicated movements would require several operations.

## Related work

The accurate placement of dental implants is important to achieve the functional and aesthetic demands. The anatomical features of the jaws limit the placement. The biggest concerns are in the molar and premolar regions. In the upper jaw, the maxilla, maxillary, and nasal cavities, and in the lower jaw, the inferior alveolaris nerve and foramen mentale set the boundaries for the implantation. In the anterior areas, the shape and the amount of available bone of the alveolar ridge may be a challenge.

Proper planning of implantation and placement of the implant are essential for successful implantation. Complications associated with implant surgery include for example bleeding, in some cases even life-threatening hemorrhages, temporary or permanent nerve injuries, malposition or displacement of implants, injury to adjacent teeth and fracture of the mandible [14–18].

VR environment can engage the viewer in a 3D space and enable evaluation of the anatomical structures from a new perspective [6]. Virtual planning improves the accuracy in dental implant placement and inserting, whether using statistic guidance or dynamic navigation [19].

Translation, rotation, scaling, and selection are common actions in virtual environments [7, 20]. Direct interaction includes grabbing the object, moving it around, and releasing the object. Direct interaction is highly intuitive in contrast to indirect interaction, like using widgets. As a downside of direct interaction, Frees et al. [21] mentioned the limited accuracy and delity of interactions. Natural hand instability and low device resolution decrease the accuracy [11].

With virtual handles, the interaction is no longer direct [22]. Hand instability limits the design of the handles, requiring them to be large enough to be easily selected, but size of handles limits their number [21].

Mendes et al. [7] discussed solutions to perform an object manipulation on a single axis, also studied by Nguyen et al. [23]. Interacting with handles restrict DoFs, while direct interaction allows use of all 6 DoFs. Direct interaction would be used for coarse transformations, while axis separation was proposed for precise transformations, like fine-tuning. Virtual handles can reduce errors, while completion time may increase. Translation and rotation may be separated also to prevent unintended transformations [11].

Hands and controllers are common interaction methods in VR, and they provide the same actions [24–26]. Controllers are widely used to manipulate virtual objects and to enhance user immersion and haptic feedback [25]. Controllers are stable and accurate, which is crucial in dental implantation. Huang et al. [25] found that users generally executed the game-like tasks more efficiently with controllers in both hands than with a controller in one hand or without any controllers. As a downside, the handheld controllers may cause arm fatigue.

## Experiment

### Implant manipulation conditions

**Fig. 1:**
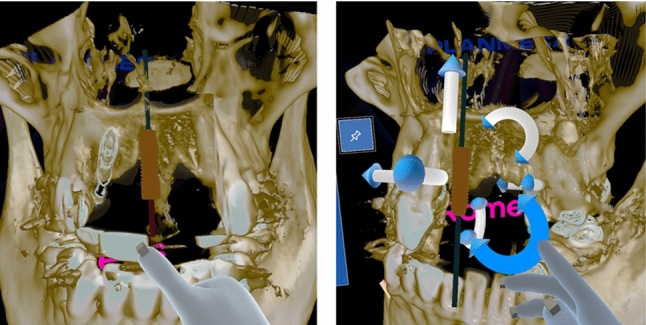
An implant without handles (left) and with handles (right). The implant could be moved by either grabbing the body or (in the second condition) also by the handles

We conducted a controlled small-scale user experiment to evaluate the usability of the virtual handles. The participants performed three dental implant planning tasks with two different conditions. In one condition, *Without handles*, the implant was manipulated by grabbing the body of the implant. In another condition, *Handles*, there were eight handles attached to the implant that enabled more careful, restricted manipulations of the position, and orientation. An implant without and with handles is shown in Fig. 1.

For the experiment, we prepared three different skull models. Skull visualizations were generated in real-time from CBCT DICOM (Digital Imaging and Communications in Medicine standard) volumetric data. Skull models were pseudonymized and may be used with permission from individuals in product development.

### Implant handles arrangement

Eight handles were located around the implant to translate and rotate the implant. The handles had a shape of an arrow. A straight arrow was for transferring the implant and a curved arrow was for rotating it. There were three straight arrows to transfer the implant along the basic axes x, y, and z. By grabbing a handle, the implant could be transferred on both directions.

The curved handles would rotate the implant around the x, y, and z axes, while the pivot point location would vary. One curved handle was perpendicular to the implant main axis to rotate the implant around that. The four curved handles that would tilt the implant were paired (one up and one down) so that grabbing the upper handle would use the apex of the implant as a pivot point and the lower handle would use the collar of the implant as a pivot point. The user could fix, for example, the apex of an implant and carefully tilt the orientation without compromising the apex location.

### Measurements

To evaluate the usability of the interaction methods in accordance with the ISO definition [27], three dimensions were investigated:

- Efficiency: Task completion time
- Effectiveness: Marking consistency
- Satisfaction: Subjective evaluations

#### Objective measures

We measured the task completion times from the moment that the implant was picked up to the moment that the participant released the implant for the last time.

For the marking consistency, we measured the positions of each implant and compared the positions in the analysis phase. We also counted the number of implant pick ups and releases.

#### Subjective measures

We asked the participants to evaluate several subjective questions and a statement (see Table 1). The participants used seven-step Likert scale for the answers, from 1 (Not-a-all) to 7 (Very). We also asked the participant to select the condition that s/he liked the best, and a short description why that specific choice.

**Table 1:** Questions (1 to 5) and a statement (6) to evaluate the subjective impressions of interaction conditions

1	How successful were you in accomplishing what you were asked to do?
2	How confident you were in your ability to use the interaction method?
3	How efficient was the interaction method to use?
4	How easy was the interaction method to use?
5	Could you imagine using the method for your daily work?
6	I needed to learn a lot of things before I could get going with this system

The facilitator observed the participants during the user study. These notes were compared to comments on the questionnaires to get overall picture of the behavior of the participant.

### Apparatus

**Fig. 2:**
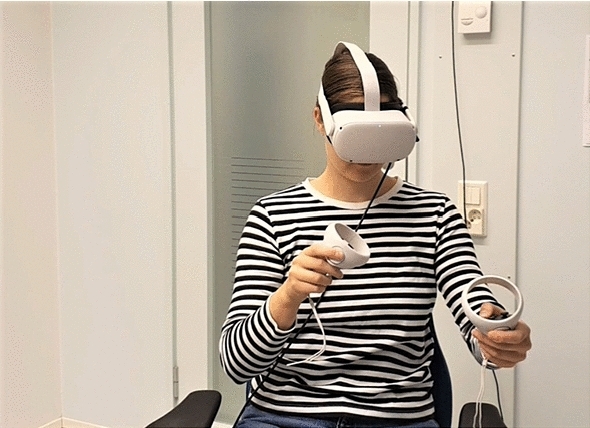
A virtual implant planning tool user with Oculus Quest 2 HMD and controllers

A VR implant planning implementation provided by Planmeca [28] was used as a foundation for the experiment software. The experimental system was based on the Oculus Quest 2 and Touch controllers (see Fig. 2). The experimental system was built using the Unity 3D software development system version 2021.1.

### Participants

We recruited four dentomaxillofacial radiologists, two seniors, with experience in dentistry for 36 and 23 years, and two residents, with experience in dentistry for 8 years. All had previous experience on the implant planning task using other planning tools and software. One senior participant did 3D implant planning daily, one resident did it weekly. The two other participants did implant planning less often. Two participants (a senior and a resident) had some experience of using VR devices, but only a few months.

### Procedure

Upon arrival, the participant was introduced to the equipment and the system functions and controls. S/he was asked to read and sign a consent form and fill in a background information form. The participant practiced the system to be able to do the tasks. When ready, the participant made three implant plans for the first condition.

For all participants, the skull models were presented in the same order. In the first skull model (model 1), the implant was positioned to the left side (patient view) of mandible bone. In the second model (model 2), the implant was positioned to right side of mandible and in the last model (model 3) to the front of the maxilla bone. The order of conditions was balanced for the experiment. Two participants did the implant planning first with *Handles* condition and then *Without handles*. The two others did it the other way around.

After three models, the participant was asked to fill a questionnaire to do a subjective evaluation of the condition. After that s/he would repeat the procedure for the second condition. After both conditions, the participant was asked to rank the conditions. The experiment, including practice and the questionnaires, took around 45 minutes per participant.

## Results

### Objective results

There was no difference in efficiency between conditions. The task completion times varied between participants and the conditions. The completion time minimums were around 40 seconds, and the maximums around 500 seconds, with no trends. The mean time was 189.3 seconds with standard deviation of 132.7 seconds.

**Fig. 3:**
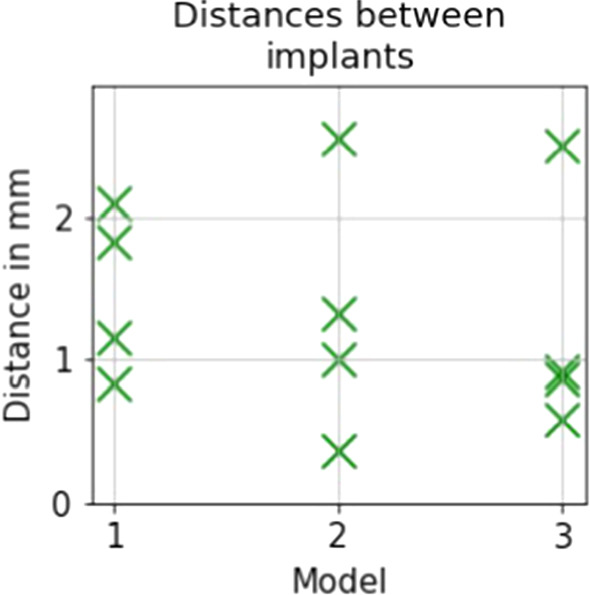
Distance between the two implants that each participant was setting into the same model (for two different conditions)

**Fig. 4:**
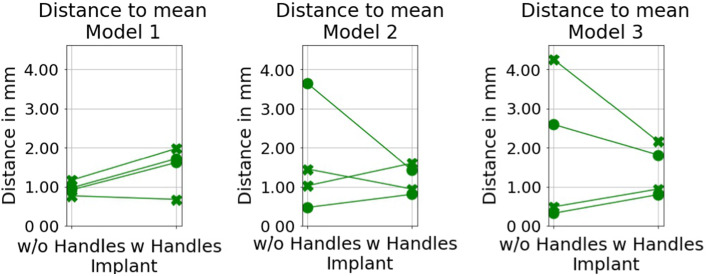
Distances of the final implant positions to the mean position computed from final positions. Connected markers on each figure are by the same participant doing the two conditions

**Fig. 5:**
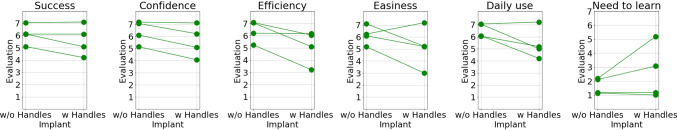
Evaluation results. A line connects the evaluations by the same participant for both conditions

We did not measure any difference in efficiency between the conditions. We recorded all the final positions of all 24 implants that were set (4 participants times, 3 models times, 2 conditions, $= 24$ positions). For the analysis of the position consistency, we computed the differences in position of the two implants that each participant set in the same model in two different conditions. The results are visualized in Fig. 3. The mean values of the differences per model (by four participants) are between 1.2 and 1.5 millimeters.

For the analysis of the position consistency between participants, we computed the mean positions from the final positions. We used the mean positions as reference locations. The distances from the final positions to the mean are shown in Fig. 4. The mean values of the distances per model and condition varied from 1.0 to 1.9 millimeters.

The numbers of implant pickups varied a lot between the participants and the conditions. The minimum numbers were 2 and 3, while the maximum was around 50, mean number was 16.8 with a standard deviation of 10.8.

### Subjective results

In the satisfaction, the subjective evaluation results show that the interaction method without handles was seen more positively. The results of the subjective evaluations are shown in Fig. 5. When asked about the success of the task (Question 1), interaction method confidence (Question 2), interaction method efficiency (Question 3) and possibility to use the method daily (Question 5), all participants evaluated *Without handles* as good as or better than *Handles*. When asked about the easiness of the interaction method (Question 4), one participant evaluated *Handles* to be easier, while the rest of the participants evaluated *Without handles* easier. The last statement was if the participant needed to learn a lot of things before working with the system, and with *Handles* more things had to be learnt.

On all measured scales, *Without handles* got better evaluation than *Handles* as indicated by mean and median values (see Table 2). Overall, both conditions were evaluated to be good, and no big differences could be found between the evaluations. The lowest mean for the condition *Without handles* was 6.0, and for *Handles* condition, it was 5.0 (excluding the last statement with contrariwise grading). The evaluation values were consistently better (higher values) for *Without handles* than for *Handles* for the five questions. For the last statement, the grade 1 means no need for learning and *Without handles* got lower values.

In the final ranking, all the four participants ranked *Without handles* to be better than *Handles*.

**Table 2:** Median and mean values of the evaluation results for the conditions

	w/o Handles	w Handles
Success		
Median	6.0	5.5
Mean	6.0	5.5
Confidence		
Median	6.5	5.5
Mean	6.25	5.5
Efficiency		
Median	6.5	5.5
Mean	6.25	5.0
Easiness		
Median	6.0	5.0
Mean	6.0	5.0
Daily use		
Median	6.5	5.0
Mean	6.5	5.25
Need to learn		
Median	1.5	2.0
Mean	1.5	2.5

## Discussion

Our experiment was a preliminary study to evaluate the use of virtual environment by professional users on their field and for that we recruited four dentomaxillofacial radiologists who did six dental implant planning tasks each. The small number of participants and tasks limits the generalizability of the results.

The task completion times varied a lot. We encouraged the participants to think aloud to get comments. As the size of a handle was smaller than an implant, grabbing a handle might have taken longer (based on Fitts’ law [29]) than grabbing an implant. Based on task completion times, the conditions are similar.

As different radiologists have slightly different views of the constraints set by the anatomical features, we do not have an unambiguous target location for the implant and some variation in placement would be expected. As shown in Fig. 3, the implant placement distances for one participant doing each model twice were very short for almost all of the participants. In half of the cases, the distance was below 1 millimeter. The implant distances between the placements by different participants varied more, see Fig. 4. In most cases (in 20 out of 24 cases), the distance to the mean position was less than 2 millimeters. This indicates that the participants performed the planning case similarly and this encourage development of this kind of VR implant planning tool.

In the evaluations, the participants indicated that it is better to do the implant placement without any handles. Multiple participants commented that *Without handles* was easy, accurate, and natural. In subjective results, almost all statements got better grades for *Without handles*. Each handle took space so that the closest handles blocked the farthest handles. In addition, grabbing a specific handle was sometimes difficult. Based on Fitts’ law [29] and Frees et al. [21], the size of the handles should not be reduced, even if the handles would then take smaller area. When grabbing the wrong handle, the implant may have moved accidentally. These issues can be fixed with further development of the handles design. The usability issues were noticed by all participants and affected the feel of use and the evaluation results.

The two modes compared here could be seen as complementing each other. While free hand is convenient in the beginning of planning, restricted movement may be required when multiple implants have been placed and their relative positions should be maintained.

The participants commented that the handles were fine and subjective results show that handles did not get poor evaluation. Two participants commented that the handles were good for fine-tuning, but large movements were difficult. When working without handles, one participant said “My hands are shaking so fine-tuning tool would be nice.” Another participant commented that s/he was surprised how accurate the implant placement was by direct interaction and that no shaking could be noticed. Participants discussed alternative fine-tuning methods for handles, which indicates that there is a need for implant fine-tuning.

An HMD presents 3D view naturally based on the position and orientation of the user’s head [5]. VR provides a realistic environment to interact with 3D implants and 3D skull model. Still the radiologists are used to 2D X-ray data, and many studies have employed the HMD to visually present this 2D medical data [30, 31]. One of the participants said that 3D environment is convenient for spatial thinking, and it is easy to understand the anatomical structure of the skull when watching and handling the 3D skull model in VR. Three out of four participants used the 2D X-ray data that was visible inside the VR environment. They used the X-ray data to verify the location of the implant, especially with the model 3 that had less bone mass. The participants commented that it is easier to study and understand the low bone mass from the X-ray data than from the 3D skull. The three participants that used the 2D X-ray data actively were more used to the implant planning task and other medical virtual planning tasks. There were also comments that the X-ray data is necessary for diagnostics.

## Conclusion

Four dentomaxillofacial radiologists performed implant planning tasks in virtual reality environment using direct and indirect object manipulation methods. The task was executed in two conditions, either the implants had virtual handles or there were no virtual handles. All the four participants ranked the direct interaction method where the implants had no virtual handles to be better than the indirect condition where implants had virtual handles. The participants noticed the advantages of the handles for fine-tuning the placement but the handle usability issues were disturbing. The direct interaction condition was commented as easy, accurate, and natural, while more learning would be required to use the virtual handles. Overall, both object manipulation methods were evaluated to be good, and the 3D virtual environment was valued for the implant planning task. The implant placements made by the radiologists were practically similar. This finding gives important information of the accuracy and potential to use this kind of VR methods in clinical work. Future research for technical and clinical validity is needed.

## Data Availability

The data is available upon request.
